# A ten year analysis of maternal deaths in a tertiary hospital using the three delays model

**DOI:** 10.1186/s12884-020-03262-7

**Published:** 2020-10-06

**Authors:** Mo’men M. Mohammed, Saad El Gelany, Ahmed Rida Eladwy, Essam Ibrahium Ali, Mohamed T. Gadelrab, Emad M. Ibrahim, Eissa M. Khalifa, Ahmed K. Abdelhakium, Hashem Fares, Ayman M. Yousef, Heba Hassan, Khaled Goma, Mahmoud H. Ibrahim, Alaa Gamal, Mohamed Khairy, Ahmed Shaban, Sahar Amer, Ahmed R. Abdelraheim, Ameirr A. Abdallah

**Affiliations:** 1 Obstetrics and Gynecology Department, Faculty of Medicine, Minia Maternity and Children University Hospital, Minia University, Elsalam, Eloboor, Maghaghaga City, Minia, Egypt; 2 Masr Elhurra Hospital, Minia Directorate of Health, Minia, Egypt; 3 Minia General Hospital, Minia Directorate of Health, Minia, Egypt

**Keywords:** Three delays model, Minia, Egypt, Maternal mortality

## Abstract

**Background:**

Reducing maternal mortality ratios (MMRs) remain an important public health issue in Egypt. The three delays model distinguished three phases of delay to be associated with maternal mortality: 1) first phase delay is delay in deciding to seek care; 2) second phase delay is delay in reaching health facilities; and 3) third phase delay is delay in receiving care in health facilities. Increased health services’ coverage is thought to be associated with a paradigm shift from first and second phase delays to third phase delay as main factor contributing to MMR.

This study aims to examine the contribution of the three delays in relation to maternal deaths.

**Methods:**

During a 10 year period (2008–2017) 207 maternal deaths were identified in a tertiary hospital in Minia governorate, Egypt. Data were obtained through reviewing medical records and verbal autopsy for each case. Then data analysis was done in the context of the three delays model.

**Results:**

From 2008 to 2017 MMR in this hospital was 186/100.000 live births. Most frequent causes of maternal mortality were postpartum hemorrhage, hypertensive disorders of pregnancy and sepsis.

Third phase delay occurred in 184 deaths (88.9%), second phase delay was observed in 104 deaths (50%), always together with other phases of delay. First phase delay alone was observed in 13 deaths (6.3%) and in 82 deaths (40%) with other phases of delay. One fifth of the women had experienced all three phases of delay together. Major causes of third phase delay were delayed referral from district hospitals, non-availability of skilled staff, lack of blood transfusion facilities and shortage of drugs.

**Conclusions:**

There is a paradigm shift from first and second phases of delay to the third phase of delay as a major contributor to maternal mortality. Reduction of maternal mortality can be achieved through improving logistics, infrastructure and health care providers’ training.

**Trial registration:**

This study is a retrospective study registered locally and approved by the ethical committee of the Department of Obstetrics and Gynaecology, Minia University Hospital on 1/4/2016 (Registration number: MUEOB0002).

## Background

Maternal mortality is an indicator of many parameters of maternal health, such as women’s status, access to care and quality of care in low resource settings [1]. Complications during pregnancy, labour and puerperium account for 303,000 women dying every year. Most deaths (99%) occur in low resource settings, especially in South Asia and sub-Saharan Africa [2]. Most deaths are preventable [3]. The maternal mortaility ratio (MMR) for Egypt in 2015 was 39/100000 [4].

Two key evidence based interventions to reduce maternal morbidity and mortality include access to Skilled Birth Attendance (SBAs) with enabling environment during labour and basic and comprehensive Emergency Obstetric and Neonatal Care (EmONC) [5]. The five direct causes of maternal mortality are obstetric hemorrhage, hypertensive disorders of pregnancy, obstructed labour, sepsis and complications of unsafe abortion, responsible for nearly two-thirds of deaths worldwide [6].

In 1994, Thaddeus and Maine’s three delays model has been introduced as an effective tool to evaluate circumstances surrounding access to and appropriateness of EmONC [7]. This model identifies barriers and intervention points during the woman’s pathway from home to hospital [8]. First phase delay which occurs at household and community level, reflects delay in deciding to seek care for pregnancy and birth complications. Second phase delay refers to delay to reach the facility and third phase delay refers to delay that happens in the health facility [9].

A recent study in Malawi showed that third phase delay was found in 96.8% of maternal deaths compared to 59.6% first phase and 39.7% second phase delays [10].

Regular annual analysis of maternal deaths in Minia university maternity hospital over a 10 years period identified different delays and subsequently addressing the findings can help to implement adequate interventions to reduce maternal mortality.

In this study, the three delays model is used to investigate different delays associated with maternal mortality that occurred in our tertiary hospital.

## Methods

### Study settings

This is an institution-based, retrospective study in Minia Maternity and Children University Hospital, Minia governorate, Egypt. Data collection was done between January 2008 and December 2017.

Minia governorate is located in Upper Egypt with an estimated population of 5.5 million in 2017 [11] . Administratively, Minia governorate is divided into nine major cities. There are three levels of health services in this governorate: primary, secondary and tertiary level. The first level is served by primary health care units while secondary care is served by nine general hospitals and the private sector. Each general hospital covers an average population of 600.000 inhabitants and has one to two ambulances to serve the surrounding primary health care units and the tertiary hospital. The nine general hospitals have capacity to offer Basic Emergency Obstetric Care (BEmONC). Tertiary level care is provided by Minia maternity university hospital. The private sector deals with the majority of cases followed by the public hospitals and all served by one tertiary hospital which is located nearly in the middle of the governorate along the 135 Kilometres road alongside the river Nile. The different layers of care are connected by a referral system based on personal relationships, private cars and ambulances managed by the general hospitals.

### Study population

All maternal deaths in Minia hospital during the study period were collected using structured form designed by the researchers to collect relevant information using multiple sources of data. These included vital registration, health facility records, burial records, and interviews with family members (verbal autopsy), physicians involved in the care during pregnancy and traditional birth attendants (TBAs).

The current research project was performed in two steps. The first stage involved detection of all maternal deaths using multiple sources. Then all deaths were examined in the second stage (using verbal autopsy, health service reports or case notes, death certificates documenting time and cause of death and interviews with family members and relatives).

To ensure data quality and to avoid missing any deaths, the research team visited registration department in the hospital once a month and again at the end of the study period, cross-checked all registers to identify any deaths not yet reported. At the end of every month, lists from the three data sources (maternal mortality meeting reports, ministry of health reports and hospital files) were compared and any duplicates removed.

Maternal death was defined according to ICD-10 as: “The death of a woman while pregnant or within 42 days of termination of pregnancy irrespective of the duration and the site of the pregnancy, from any cause related to or aggravated by the pregnancy or its management but not from accidental or incidental causes” [12].

Trained research staff visited all households in which maternal death had occurred to interview all persons (including relatives, TBAs, primary health care staff and neighbours) who had knowledge of the woman’s illness and death using a standard verbal autopsy questionnaire designed by the ministry of health according to WHO format [13]. They prepared a separate report for each case of maternal death that has been reviewed by an expert panel to assess its underlying cause of death and factors associated with each case.

### Data collection

Data included age, parity, booking status, referral source, possible cause of death and substandard care factors, time of arrival in the emergency room, as well as time of treatment initiation with a medical or surgical intervention, time of death, care received in the periconceptional and perinatal period, place of birth, decisions taken by health care providers involved in care (e.g. obstetricians, general practitioners?, midwives? TBAs, ….), journey from home to health facility, referral status, and impression of the care perceived from the relatives about its quality provided in the health facility.

Each case was assigned with a single underlying cause of death. For each maternal death, data sheets were created using all documents, including any medical records, all verbal autopsy information, medical personnel interviews and/or information obtained through a maternal death review conducted at the health facility.

Data were reviewed by a committee of experts composed of seven Ob/Gyn consultants affiliated to Minia University in addition to a representative from the maternal mortality counsel of the ministry of health. The committee reviewed the circumstances of each death to determine: 1) underlying cause of death which initiated the chain of events that ultimately led to death of the woman, 2) Any evidence suggesting delay at various levels [7]. Data regarding first and second phase delay were obtained from interviews with patients’ relatives whereas that regarding the third phase delay was taken from the case files and interviews of the physicians involved in the care.

A note was made of substandard care factors which could have contributed significantly to maternal deaths. Substandard care was defined as care received by the woman falling below the standard that should have been offered to her in accordance with the agreed local protocols in our institution.

### Application of the three delays model

The second phase delay involved delay in transfer from different health facilities and numerous referrals while delay in seeking medical care due to family or community factors was considered as a first-phase delay. Delay in reaching the health facility with the capacity to provide basic emergency obstetric care services is a second-phase delay. Women who faced delay in the process of referral to the second health facility were known as second phase delay. If evidence indicates delay in providing sufficient health care inside the health facility (i.e. general hospitals, private sectors or Minia teriary hospital), it is classified as a third phase delay. Delay was described on the basis of evaluating the individual story of each woman since her arrival at the original health facility.

All data were independently checked by the research team. Key themes were identified and used for the creation of a structured analytical form to define each phase of delay: first phase delay (lack of financial support, unavailability of husband, lack of knowledge of obstetric risks, social problems, previous uncomplicated pregnancy, previous poor health facility experience or perceived poor quality of care in the health facility, avoiding admissions). Second phase delay (Absence of a local health facility, travel costs, bad road conditions, long distance to the nearest hospital, availability of TBAs), third phase delay (long waiting period prior to receipt of treatment (more than 30 min from the time of arrival to the time of assessment or care), lack of equipment and resources, unavailability of health care providers, absecne of experienced staff, lack of infrastructure, shortage of medicines and necessary medications, incorrect risk assessment, inaccurate diagnosis and treatment, lack of a referral system, lack of treatment policies). In one particular instance, there might have been one or more delays. Then, all cases were examined jointly. In the event of disagreement, joint meetings with analysis of the issues of disagreement were held before consensus was reached.

### Statistical analysis

Data were analysed by computer software, SPSS version 20, and results presented as frequencies and percentages.

## Results

A total of 207 maternal deaths were identified in the study setting, out of 107,444 deliveries and 110,766 live births, during the period of study corresponding to a facility based maternal mortality ratio of 186 deaths per 100.000 live births (95% CI: 162–213).

Over 10-years (2008–2017), the women who died, had a mean age of 28.8 ± 6.2 years with a range between 18 and 40 years. Only 15 (7.2%) were < 20 years and 10 (4.8%) were ≥ 40 years. Most women were multiparous (159; 76.8%) while 48 (23.2%) were primiparas and 92 women (44.4%) had no formal education.

Almost three quarters (152; 73.4%) of the maternal deaths had attended ≥4 ANC visits, while only 15 women (7.2%) did never attend ANC. Most of them (170 women) (80%) received ANC in the private sector, while only 20 women (9.9%) were booked in primary health care facilities. In the study setting hospital only six women (2.9%) were booked.

The majorty of women died within 24 h after birth (85 women, 41%). About one third of women died after the first 24 h till the end of puerperium (60 women, 29%).Thirty three women (15.9%) died during birth and 29 women (14%) died during pregnancy.

Most frequent causes of direct maternal mortality were postpartum hemorrhage (24.6%), hypertensive disorders of pregnany (19.8%) and sepsis (9.7%). Most common cause of indirect maternal mortality was cardiac diseases (6.3%) (Table 1).

**Table 1:** Underlying cause of death

Cause	N (%)
**Direct maternal deaths**	**162 (78.3%)**
Postpartum haemorrhage	51 (24.6%)
Hypertensive disorders of pregnancy	41 (19.8%)
• Eclampsia	21 (10.1%)
• Severe preeclampsia	20 (9.7%)
Sepsis	20 (9.7%)
Thromboembolism	12 (5.8%)
Anaesthetic complications	9 (4.3%)
Uterine rupture	7 (3.5%)
Placenta previa	5 (2.4%)
Placental abruption	4 (1.9%)
Hyperemesis gravidarum	4 (1.9%)
Suspected amniotic fluid embolism	3 (1.4%)
Ectopic pregnancy	2 (1.0%)
Molar pregnancy	2 (1.0%)
Acute uterine inversion	2 (1.0%)
**Indirect maternal deaths**	**42 (20.3%)**
Cardiac disease	13 (6.3%)
Hepatic diseases	10 (4.9%)
Chronic chest diseases	6 (2.9%)
Malignancy	5 (2.4%)
Chronic renal diseases	5 (2.4%)
Idiopahic thrombocytopenic purpura	3 (1.4%)
**Other causes**^a^	**3 (1.4%)**
**Total**	207 (100%)

^a^Other causes include two cases of ovarian hyperstimulation syndrome (OHSS) and one case with anaphylactic reaction

Almost one third of the women who eventually died, were referred from general hospitals, one quarter was directly admitted to the tertiary hospital and one quarter came from home after being managed by TBAs (home care), while the private sector referred one fifth (Table 2). Referred women to the tertiary hospital from the private and/or public hospitals were primarily attended by TBAs and/or primary health care physicians.

**Table 2:** Place of care before death

Place of care before death	N (%)
**Private place**	**40 (19.3%)**
**Home care**	**50 (24.2%)**
**General hospital**	**66 (31.9%)**
**University hospital**	**51 (24.6%)**

Time between admission and death ranged from 0 to 572 h with a median of 14 days (IQR 10.2 days). Fourteen women died immediately on arrival and another fourteen died within 2 h after failed resuscitation, while fifty-seven women died within 24 h of admission.

### Phases and frequencies of delays

**Fig. 1:**
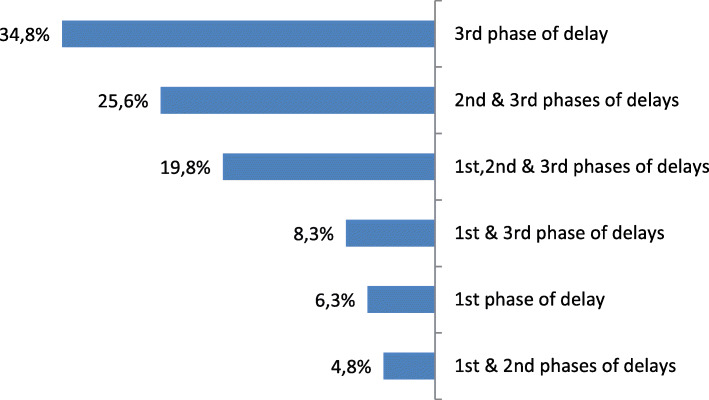
Phases of delay

The majority of women suffered from multiple phase delays (58.9%), while the rest (41.1%) had separate forms of delays (first & third phase). Second phase delay never occurred on its own (Fig. 1). Third phase delay was present in almost 88.9% either alone or in conjunction with other phases of delay (Table 3). Reasons of referral to the tertiary hospital are presented in Table 4.

**Table 3:** Three phases of delay (*n* = 207)

Phase of delay	Causes	N (%)
**First phase delay** (82 cases) (39.6%)	**Lack of awareness of obstetric complications**	**40 (19.3%)**
	**Low status (women not financially empowered and/or non availability of husband).**	**30 (14.5%)**
	**Uneventful previous home births.**	**30 (14.5%)**
	**Previous bad experience of health care**	**20 (9.7%)**
	**Perceived poor quality of care in healthcare facilities**	**25 (12.1%)**
	**Low family income**	**28 (13.5%)**
	**Visited TBAs or unexperienced physician.**	**45 (21.7%)**
	**Long waiting in a healthcare facility**	**10 (4.8%)**
	**Domestic violence**	**10 (4.8%)**
**Second phase delay** **(104 cases)** **(50.2%)**	**Late referral to the tertiary hospital**	**50 (24.2%)**
	**Multiple referrals**	**39 (18.8%)**
	**Home –Health facility Long distance .**	**29 (14%)**
	**Lack of a health facility in the area**	**50 (24.2%)**
	**Poor or inaccessible road conditions.**	**10 (4.8%)**
	**High costs of transport**	**5 (2.4%)**
**Third phase delay** **(184 cases)** **(88.9%)**	**Inadequate referral system**	**100 (48.3%/)**
	**Senior staff unavailable**	**110 (53.1%)**
	**Shortage of equipment and supplies**	**60 (29%)**
	**Lack of competence on EmOC in health facilities**	**110 (53.1%)**
	**Wrong assessment (of risk, diagnosis, treatment)**	**63 (30.4%)**
	**Lack of treatment guidelines**	**40 (19.3%)**
	**Shortage of trained staff**	**67 (32.4%)**
	**Long waiting time before treatment in health facility**	**14 (6.8%)**

*TBAs* Traditional birth attendants, *EmOC* Emergency Obstetric Care

**Table 4:** Reasons of referral to the tertiary hospital (N 156 = 75.4%)

Reason	N (%)
**Non -availability of blood**	**110 (53%)**
**Non-availability of intensive care unit (ICU)**	**70 (33.8%)**
**Non-availability of anaesthetists**	**64 (30.9%)**
**Non-availability of skilled personel**	**33 (15.9%)**
**Shortage of equipment and supplies**	**134 (64.7%)**

## Discussion

In the current study, the MMR from 2008 to 2017 was 186/100.000 live births which is remarkably higher than has to be achieved by SDGs [14]. This hospital based MMR is almost five times higher than the national MMR for Egypt which was 39/100000 live births in 2015 [4]. This may be explained by the fact that Minia university hospital is a tertiary referral centre and complicated cases in the whole Minia governorate are referred to the hospital. A hospital based study about maternal mortality was conducted in Assiut governorate (Egypt) which is very near to Minia governorate with similar circumstances. MMR in Women Health Hospital, Assiut University, Egypt between 2009 and 2014 was 180/100.000 which is very similar to what was reported in our study [15].

All three phases of delay were present in 40 women (19.3% of maternal deaths). The most frequent delay occurred in the third phase. This occurred alone in 34% and in 88.9% in conjunction with the other two phases of delay. A similar study in Malawi found that third phase delay was encountered in 96.8% of maternal deaths [10]. Poor outcome has been shown to be associated with each phase of delay [16]. Substandard care was present in all three phases of delay in all health facilities [7].

All contributing factors to maternal mortality in our study are similar to findings in other studies in Malawi, Nigeria, Zambia, the Gambia, Ethiopia and Indonesia [10, 17–22].

A well-functioning health system should provide effective and efficient care through skilled health care providers in well-equipped health facilities with essential medications and equipment. That has been prescribed by Adegoke et al. as “enabling environment” [23].

In a study conducted in Mozambique’s Maputo Province, covering 564 surviving women and 71 maternal deaths, second phase delay occurred in 21.3% and third phase delay in 69.7% of cases [24, 25]. In the same study, these delays followed the woman’s path throughout the referral system from admission to a peripheral health facility on to the terminal referral hospital [25].

Sometimes instability in the security situations in Egypt led to second phase delay in referral from home to hospitals. In Malawi, fuel shortage led to delay in referral to health facilities [10, 26]. Lack of proper referral system has been reported as a cause contributing to 25% of maternal deaths in some studies [26]. In the Gambia, use of ambulances for others purposes forced women to seek alternatives and to pay for transport [27]. Absence of emergency obstetric care also has been reported to be a major cause of maternal mortality with a frequency reaching up to 60% of maternal deaths in some studies in low and middle income countries [28–30]. Facilities providing EmONC may be extremely far from women’s homes. Maternity waiting homes (MWH) have been proposed as strategy to overcome inaccessability [31]. MWHs are places with easy access to hospital in which women with high risk pregnancies near term await birth to assure easy admission to hospital once labour starts or complications like hemorrhage arise. We think that MWHs could be beneficial in our locality as the “ health facility’s long distance” was implicated in 14% of maternal deaths in our study.

In our study, first and second phase delays were experienced less frequently than third phase delay. Poor quality of care may influence women’s decisions to access care [32].

Remarkably the great majority of women who died had attended some form of ANC, while only 7.2% did not attended even once. This is paradoxical to other studies, in which most maternal deaths occurred in unbooked women [33–35]. This paradox is in agreement with official reports of the Egyptian ministry of health and may be explained from the point of view that women with high risk pregnancies receive ANC while women with low risk pregnancies do not book for ANC. An additional factor is the lack of standard ANC with its poor quality.

Women in our study still faced important risks of dying, even when they arrived directly in the health facility, being in a healthier status [5]. Third phase delay was implicated in the majority of maternal deaths in our study.

### Strengths and limitation

Being the first study in Egypt to apply the three phases delay model to analyse maternal mortality in a 10 years period in a large tertiary hospital is one of the strengths of this study. This can be replicated in other governorates in Egypt, which can provide policy managers with data to plan appropriate health interventions for reduction of maternal mortality. Another strong point is the analysis by the expert group that helped to reduce inconsistencies in the data from the files.

There were limitations as well. Limiting data to one health facility, being a tertiary hospital in one Egyptian governorate limits generalization to the rest of the country due to different geographic patterns even in the same country. Also, inclusion of a control group of women who survived, would possibly have clarified other determinants of maternal mortality. Although many sources for data collection were available, the quality of hospital and health centre registers and medical charts were poor, especially for data about ANC. Another limitation to take into account is the fact that this study is “ institution based”, so maternal deaths at home or in general and private hospitals are not studied here.

Regarding implications of the current study, there is an urgent need for reinforcing the referral pathways between hospitals and optimising the hospital needs with equipment to provide timely and adequate care in a friendly manner with respect to women as prerequisites to save the lives of women and newborns.

## Conclusions

The current study provides policy makers with clues to initiate interventions and recommendations to reduce maternal mortality at different levels of care, also in similar national and international settings. There is a paradigm shift from first and second phases of delay to the third phase of delay as a major contributor to maternal mortality. Inadequate referral system, senior staff unavailability and lack of competence on basic and comprehensive EmOC are implicated in 50% of maternal deaths, all of them modifiable factors. This will need urgent improvements in the whole maternal health system.

## Data Availability

The datasets used and/or analyzed during the current study are available from the corresponding author on reasonable request.

## Abbreviations

ANC: Antenatal Care
BEmOC: Basic Emergency Obstetric Care
EmOC: Emergency Obstetric Care
EmONC: Emergency Obstetric and Neonatal Care
HTN: Hypertensive disorders
IQR: Interquartile range
MMRs: Maternal mortality ratios
OHSS: Ovarian hyperstimulation syndrome
SBAs: Skilled Birth Attendants
TBAs: Traditional Birth Attendants
MWH: Maternity waiting home
